# High-throughput screening and evaluation of repurposed drugs targeting the SARS-CoV-2 main protease

**DOI:** 10.1038/s41392-021-00763-5

**Published:** 2021-09-29

**Authors:** Yan Li, Jinyong Zhang, Zilei Duan, Ning Wang, Xiangcheng Sun, Yanjing Zhang, Li Fu, Kaiyun Liu, Yongjun Yang, Shulei Pan, Yun Shi, Hao Zeng, Gang Guo, Ren Lai, Quanming Zou

**Affiliations:** 1 West China Biopharm Research Institute, West China Hospital, Sichuan University, Chengdu, Sichuan 610041 China; 2 National Engineering Research Center of Immunological Products, Department of Microbiology and Biochemical Pharmacy, College of Pharmacy, Army Medical University, Chongqing, 400038 China; 3 Key Laboratory of Animal Models and Human Disease Mechanisms of Chinese Academy of Sciences/Key Laboratory of Bioactive Peptides of Yunnan Province, Kunming Institute of Zoology, Chinese Academy of Sciences, Kunming, Yunnan 650223 China; 4 Kunming National High-Level Biosafety Research Center for Non-Human Primates, Kunming Institute of Zoology, Chinese Academic of Sciences, Kunming, Yunnan 650107 China; 5 The Research Core Facility, West China Hospital, Sichuan University, Chengdu, Sichuan 610041 China; 6 Medical Research Center, Southwest Hospital, Army Medical University, Chongqing, 400038 China

**Keywords:** Drug screening, Target validation

**Dear editor**,

To date, a number of clinically approved drugs have been evaluated for potential to treat coronavirus disease 2019 (COVID-19), such as lopinavir/ritonavir, hydroxychloroquine, cobicistat, and darunavir. Some of these drugs have been proven to be effective in vitro; however, clinical trials showed that none of these compounds led to a significant improvement in symptoms or length of hospitalization. Thus, it is essential and more reliable to start from a defined target to identify candidate drugs.

The severe acute respiratory syndrome coronavirus 2 (SARS-CoV-2) main protease M^pro^, also known as 3CL protease, is one of the best-characterized drug targets among coronaviruses. In the current study, structure and sequence alignment based on two different structures of SARS-CoV-2 M^pro^ (PDB ID: 6lu7 and 6m2q) showed an obvious change between Ser’46/CA and Leu’167/CA, which indicated that the substrate-binding pocket of M^pro^ exhibits a certain extent of flexibility (Supplementary Fig. S1). Therefore, we developed a multiple cross-docking strategy with these two structures, to perform a computer-based high-throughput virtual screening of possible inhibitors from a drug database and our in-house automatic processing scripts using AutoDock Vina software (Supplementary Fig. S2). A total of 108 molecules with a docking score of < −8.0 kcal/mol were found against both of these structures. Then, 37 molecules with molecular weights between 330 and 700 g/mol with respect to the pocket volume and pKa values > 12 were selected. After that, we focused on 11 antiviral, antibacterial, and target-oriented antitumor drugs (Supplementary Table 1). Moreover, several previously reported drugs were used for reference, such as GC376,^1^ lopinavir, nelfinavir, and darunavir.

Full-length SARS-CoV-2 M^pro^ was expressed and purified based on its coding sequence (GI: 1897214688) and the affinity of each of the screened candidates for M^pro^ was detected by the surface plasmon resonance (SPR) technique. The response units were measured with a Biacore instrument using a gradient concentration of the small molecules that interacted with M^pro^; then, the receptor-ligand binding affinity was measured and reported as the equilibrium dissociation constant (*K*_D_) based on curve fitting under steady-state analysis. Six drugs exhibited excellent binding affinity for M^pro^, including entrectinib, indinavir, cloxacillin, dolutegravir, saquinavir, and enasidenib, with *K*_D_ values of 55 μM or below (Fig. 1a).

**Fig. 1:**
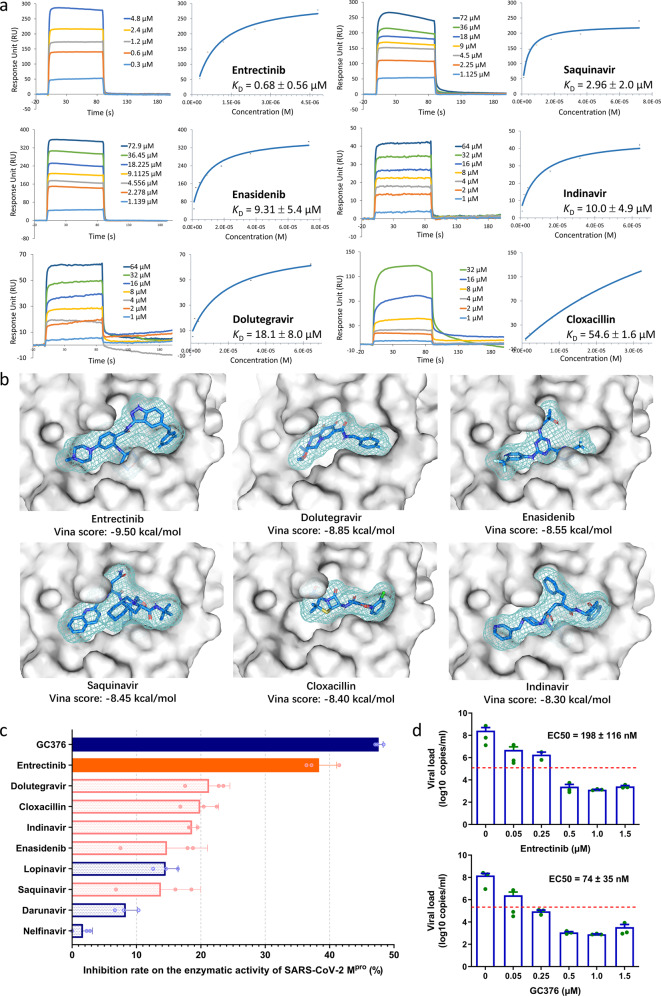
Screening and evaluation of repurposed drugs targeting SARS-CoV-2 M^pro^. **a** SPR plots of the six small molecules binding to M^pro^. The real-time binding kinetic profiles of each molecule at a gradient of concentrations were recorded in the sensorgrams with a Biacore system and then the fit curves were constructed by using the steady-state affinity model. These experiments were repeated twice. **b** Molecular docking poses of the screened candidate drugs in the substrate-binding pocket of M^pro^. The relevant parameters of each molecule are listed below each image. **c** Comparison of the inhibitory efficacies of the identified drugs on the enzymatic activity of M^pro^. In a total volume of 280 μL, each reaction component was added triplicate wells with the following final concentrations: 20 μM drug, 1.5 µM M^pro^ and 10 μM substrate (Dabcyl-KTSAVLQ/SGFRKME-Edans). RFU values were measured every 10 mins with excitation at 340 nm and emission at 510 nm for 3 ~ 6 h. The inhibition rate was calculated based on the change in the average RFU value between each drug treatment and the DMSO control. Results are shown as the means ± SD after two consistently performed independent assays. **d** Antiviral activity of entrectinib and GC376. Vero E6 cells were infected with SARS-CoV-2 at an MOI of 0.01 for 2 h and then treated with different concentrations of entrectinib or GC376. Forty-eight hours after infection, the supernatants were collected and viral copies were detected by qRT–PCR. Data are shown as the means ± SEM with *n* = 3 biological replicates. A cutoff line of EC50 is drawn at half of maximum effect by using a dashed line in red. These experiments were repeated twice.

Next, mutagenesis studies were performed to confirm the specificity of the interaction between the screened molecules and SARS-CoV-2 M^pro^. First, an in-depth analysis of the substrate-binding pocket of M^pro^ identified seven residues that may interact with these molecules, including His41, Asn142, Cys145, His164, Met165, Asp187, and Gln189. Among them, His41, Cys145, His164, and Asp187 have been reported as potential catalytic residues.^2^ Then, a mutant of M^pro^ was designed in which all seven of these residues were replaced with Ala. Computer-aided simulation indicated that the main structure of this mutant remained stable, except for the substrate-binding pocket (Supplementary Fig. S3b) in comparison with the wild-type M^pro^ (Supplementary Fig. S3a). Finally, SPR examination showed that the mutant enzyme lost or decreased its capacity to recognize the abovementioned molecules (Supplementary Fig. S3c), indicating that these molecules bind specifically to the active pocket of M^pro^ and further confirming that the method used for drug screening is reliable.

Then, we examined the molecular docking poses of the identified drugs with a high affinity for SARS-CoV-2 M^pro^. Typically, there are four types of docking conformations, which are represented by fluoroquinolone antibiotics, HIV protease inhibitors, kinase inhibitors and HIV integrase inhibitors (Fig. 1b). Regardless of whether the pocket was entirely filled, various docking conformations showed satisfactory affinities by SPR examination. However, the SPR affinity results did not show a complete association with the docking conformation results.

Furthermore, to investigate the inhibitory efficacies of these drugs on the enzymatic activity of M^pro^, we determined the changes in relative fluorescence units (RFUs) when SARS-CoV-2 M^pro^ acted on the Förster resonance energy transfer substrate Dabcyl-KTSAVLQ/SGFRKME-Edans under the influence of drugs or not. An effective inhibitor would significantly interrupt M^pro^ cleavage of the quenched fluorogenic substrate and thus suppress the increase in RFU value. First, we evaluated the efficacies of the reference molecules, including GC376, lopinavir, and nelfinavir. Among them, GC376, a US Food and Drug Administration-unapproved inhibitor of 3CL proteases, expressed the highest inhibition efficacy. In contrast, lopinavir, darunavir, and nelfinavir exhibited either no or only weak inhibitory effects. This result provided critical evidence to interpret the lack of antiviral activity of these drugs in clinical trials, although they exhibited anti-SARS-CoV-2 effects in previously reported cellular experiments. Based on our results, several clinically approved drugs screened in this study could be considered candidate drugs, because they exhibit a definite affinity and inhibitory efficacy on the enzymatic activity of SARS-CoV-2 M^pro^ (Fig. 1c). Among them, entrectinib is an orally bioavailable inhibitor of multiple protein kinases (TRK, ROS1, and ALK) with antineoplastic activity^3^ and this study suggested that entrectinib also has outstanding inhibitory efficacy against SARS-CoV-2 M^pro^ that is comparable to that of GC376.^1^ Moreover, cloxacillin, indinavir and dolutegravir also showed moderate inhibition of the enzymatic activity of SARS-CoV-2 M^pro^.

In contrast to previous methods,^4^ in the inhibitory assay in this study, after testing a gradient of concentrations of M^pro^ (1.5, 0.5 and 0.25 µM), we performed the enzymatic inhibitory experiment with a higher concentration of M^pro^ (1.5 µM) and a longer reaction time to improve the signal-basal state rate and decrease the background noise. Thus, the obtained inhibitory rates were relative values, generating more reliable comparative data for us to determine the different inhibitory performances of these drugs on the activity of SARS-CoV-2 M^pro^. The results showed that not all of the high-affinity drugs had significant inhibitory efficacy; thus, functional experiments are critical for the final validation. For instance, we detected the nelfinavir had a high affinity for M^pro^ (*K*_D_ = 2.36 µM) and its EC50 value (the drug concentration that inhibits half of the maximal efficacy) was reported to be 1.13 µM in the cellular experiment,^5^ but it finally failed to block the enzymatic activity of M^pro^ in this functional assay. Then we measured the IC50 (the drug concentration that inhibits half of the enzymatic activity) of entrectinib identified in this study. An enzymatic inhibitory experiment with 200 nM M^pro^ was performed by using a concentration gradient of entrectinib and the reference compounds. Based on the fitting curves of inhibition (Supplementary Fig. 4), the IC50 value of entrectinib was 10.6 µM, which was comparable to that of GC376 (13.8 µM) but significantly lower than that of nelfinavir (41.5 µM). We noticed that the IC50 values were inconsistent in different reports, so it is very important to set an internal reference in each experiment, such as GC376 in our study. It testified that the comparative data are more reliable than a single value to evaluate the activity of compounds.

Finally, the antiviral activity of entrectinib was examined in Vero E6 cells infected with SARS-CoV-2 at the biosafety level-3 laboratory of the Kunming High-level Biosafety Primate Research Center, Yunnan, China. Infected cells were treated with different concentrations of drugs at safe doses (Supplementary Fig. S5) for 48 h and viral RNA copies were detected by a quantitative reverse transcriptase PCR assay using SARS-CoV-2-specific primers. The EC50 values of entrectinib and GC376 were determined to be 198 and 74 nM, respectively, as calculated by the fit curve (Fig. 1d). This result demonstrated that entrectinib exhibited promising anti-SARS-CoV-2 activity at the cellular level.

Taken together, based on a virtual drug screening workflow followed by experimental affinity and inhibitory efficacy evaluations, we identified several drugs that not only have a definite docking conformation and binding affinity to SARS-CoV-2 M^pro^ but also exhibit a stronger ability to suppress its activity than previously reported drugs, such as lopinavir, darunavir, and nelfinavir. This study provides more solid evidence at the molecular level to interpret the differences and mysteries between previous cellular experiments and clinical trials for antiviral treatment of COVID-19. Our results suggest that some clinically approved drugs, such as entrectinib, may serve as promising candidates to treat SARS-CoV-2 infection.

## Supplementary information

All of the Supplementary_Materials

Data Set 2

Data Set 4

Data Set 6

Data Set 8

Data Set 10

Data Set 12

Data Set 14

Data Set 16

Data Set 18

Data Set 20

Data Set 22

Data Set 25

Data Set 27

Data Set 29

Data Set 31

Data Set 33

Data Set 35

Data Set 37

Data Set 39

Data Set 41

Data Set 43

Data Set 45

Data Set 1

Data Set 3

Data Set 5

Data Set 7

Data Set 9

Data Set 11

Data Set 13

Data Set 15

Data Set 17

Data Set 19

Data Set 21

Data Set 23

Data Set 24

Data Set 26

Data Set 28

Data Set 30

Data Set 32

Data Set 34

Data Set 36

Data Set 38

Data Set 40

Data Set 42

Data Set 44

Data Set 46

## Data Availability

The data used and analyzed in this study are available in the main text and the Supplementary Materials. Any other raw data that support the findings of this study are available from the corresponding author upon reasonable request.
