# Supplementary material for: High-throughput screening and evaluation of repurposed drugs targeting the SARS-CoV-2 main protease
Source: Signal Transduct Target Ther. 2021 Sep 29;6:356. doi: 10.1038/s41392-021-00763-5 (PMC8479016; doi:10.1038/s41392-021-00763-5)
Supplement: Supplementary file 1 — All of the Supplementary_Materials [file 41392_2021_763_MOESM1_ESM.docx]

Supplementary Materials for

High-throughput Screening and Evaluation of Repurposed Drugs Targeting SARS-CoV-2 Main Protease

Yan Li^1#^, Jinyong Zhang^2#^, Zilei Duan^3,4#^, Ning Wang^1^, Xiangcheng Sun^1^, Yanjing Zhang^5^, Li Fu^5^, Kaiyun Liu^1^, Yongjun Yang^6^, Shulei, Pan^1^, Yun Shi^1^, Hao Zeng^2^, Gang Guo^1*^, Ren Lai^3,4*^, Quanming Zou^2*^

Correspondence to: Gang Guo (guogang7001@163.com)

Lai Ren (rlai@mail.kiz.ac.cn)

Quanming Zou (qmzou2007@163.com)

**This PDF file includes:**

Materials and Methods

Figures. S1 to S5

Table S1

Captions for Data S1

**Other Supplementary Materials for this manuscript include the following:**

Data S1.zip

Materials and Methods

**Data resources**

The biological sequences of SARS-CoV-2 (accession no.: NC_045512.2) were obtained from the NCBI database. The 3D structures and sequences of SARS-CoV-2 M^pro^ (6lu7, 6m2q) were downloaded from the PDB database ^1^. The database with 10695 small molecule drugs was obtained from DrugBank (version 5.1.5) ^2^.

**Reagents**

The drugs used in this study were purchased from TargetMol, USA, for the following experiments.

**Molecular docking**

The crystal structures of the M^pro^ monomer of SARS-CoV-2 were prepared for the target proteins after removing the unrelated molecule, followed by removing water, adding hydrogen and computing charges by using AutoDockTools ^3^. The binding coordinates were located by a grid box against the active pocket. High-throughput autodocking was performed by using multi-threaded tasks by our in-house script based on AutoDock Vina ^4^, and potential molecules were screened out by a Perl program developed by us, running at Ubuntu server (version: 19.10-live-server-amd64) with an AMD Ryzen 3950x (16-core, 32-thread) processor. Moreover, another docking affinity prediction was performed by SeeSAR, according to the general operating process (version 9.2; BioSolveIT GmbH, Sankt Augustin, Germany, 2019, www.biosolveit.de/SeeSAR). The 3D molecule images were visualized by PyMOL v2.3^5^.

**Cloning, expression and purification of wild-type** M^pro^

The nucleotide fragment encoding full-length M^pro^ in the orf1ab sequence (YP_009724389.1) was codon-optimized for usage in the Escherichia coli (*E. coli*) expression system with JAVA Codon Adaptation Tool. The optimized sequence was synthesized and cloned into an expression vector derived from the pGEX-6p-1 plasmid (Novagen) via BamH1 and XhoI restriction sites by Sheng Gong Biological Engineering (Shanghai, China). *E. coli* BL21 (DE3) cells (Invitrogen) transformed with the recombinant plasmid pGEX-6p-1- M^pro^ were grown at 37°C in 2 L of Luria-Bertani medium until the OD600 reached 0.6, and then, 0.2 mM IPTG was added to induce the expression of recombinant protein at 16°C overnight. The bacterial pellets were resuspended in PBS and disrupted by ultrasonication. The supernatant was harvested by centrifugation and mixed with glutathione Sepharose 4B agarose (GE Healthcare, NJ, USA) and incubated for 3 hours at 4°C. Then, the beads were washed with PBS, and preScission protease (GE) was added to cleave the GST tag. The supernatant containing the GST-free M^pro^ was collected, concentrated and applied onto a HiLoad 16/60 Superdex 200 prep-grade gel filtration column (GE Healthcare, NJ, USA) equilibrated with 20 mM Tris–HCl pH 7.5, 150 mM NaCl, 1 mM DTT, 1 mM EDTA and 5% glycerol. The peak fractions corresponding to the recombinant protein were pooled for further studies.

**Site-directed mutagenesis**

An M^pro^ mutant was designed by substituting 7 amino acids (41H/A, 142N/A, 145C/A, 164H/A, 165M/A, 187D/A, 189Q/A) in the active pocket, followed by a mutagenesis analysis using the wizard of PyMOL. Then, the recombinant plasmid pGEX-6p-1-m M^pro^ encoding the M^pro^ mutant was synthesized by Sheng Gong Biological Engineering (Shanghai, China). The protocol used for expression and purification of the mutant protein was the same as that used for wild-type M^pro^.

**Surface plasmon resonance (SPR) assay**

All SPR experiments were carried out on a Biacore X100 (GE Healthcare, Uppsala, Sweden) with active temperature control at 25℃ following the manufacturer’s protocols. For protein immobilization, 100 μl of 50 μg/ml M^pro^ protein in sodium acetate buffer (pH 5.0) was prepared for amino coupling onto channel 2 of a CM5 sensor chip (GE Healthcare), and channel 1 was used as the reference flow cell [1]. Candidate chemical molecules were diluted in a linear gradient with running buffer (0.02 M phosphate buffer, 2.7 mM KCl, 137 mM NaCl, 0.05% surfactant P20, pH 7.4) and made to flow across immobilized M^pro^ protein for 150 s at a flow rate of 10 μl/min (association), followed by dissociation in the running buffer for 90 s. The resulting data were fitted to a 1:1 binding model using Biacore Evaluation Software (GE Healthcare).

**Enzymatic activity inhibition assays**The M^pro^ activity and inhibition assays were refined from the previous descriptions ^6-8^. A FRET based substrate with a natural cleaving site of M^pro^ between the NSP4-NSP5 junction from SARS-CoV-2 (Dabcyl-KTSAVLQ/SGFRKME-Edans) was synthesized (Genscript Inc, China). At each well in black 96-well flat-bottem plate, 10 µl drug in DMSO was respectively pre-incubated with 120 µl SARS-CoV-2 M^pro^ in pH 6.5 reaction buffer (20 mM HEPES, pH 6.5, 120 mM NaCl, 0.4 mM EDTA, 4 mM 1,4-dithiothreitol (DTT) and 20% glycerol) for 30 min at 37 °C, 150 µl FRET substrate in pH 6.5 reaction buffer was added to the above mixture to initiate the reaction at a final concentration of 20 µM drug, 1.5 µM M^pro^, 10 µM FRET substrate. In the positive control, 10 µl of DMSO replaced the drug for detecting original enzyme activity. The florescence baseline was checked using negative control with 10 µl DMSO, 120 µl buffer and 150 µl substrate; The reaction was monitored with filters for excitation at 340 nm and emission at 510 nm every 10 min for 3~6 h at 37 °C by using Synergy H1 Hybrid Multi-Mode Reader (BioTek, USA). By using Microsoft Excel, the RFU increment was calculated by the RFU at reaction end subtracting the start one at linear region in each well and the florescence baseline, the mean was calculated in triplicate wells. The inhibition rate of each drug was computed by the formula: 1- RFU increment of drug interfering group/RFU increment of original enzyme activity.

**Antiviral activity assay**

Vero E6 cells were infected with SARS-CoV-2 at an MOI of 0.01 for 2h, which were then treated with different concentrations of Entrectinib or GC376. 48 h after infection, supernatants were harvested and virus RNA copies were detected by qRT-PCR assay using the SARS-CoV-2-specific primers. Primer sequences used for quantification of the SARS-CoV-2 included forward primer, 5'-GGGGAACTTCTCCTGCTAGAAT-3' and reverse primer, 5'-CAGACATTTTGCTCTCAAGCTG-3'. Probe sequence used for quantification was 5'-FAM-AGCCGCCGCCTGGTCAACTCG-TAMARD-3'. Data are shown as mean ± SEM, n=3 biological replicates, these experiments were repeated twice.

**References**

1 Jin, Z. *et al.* Structure of Mpro from SARS-CoV-2 and discovery of its inhibitors. *Nature*. **582**, 289-293, (2020).

2 Wishart, D. S. *et al.* DrugBank 5.0: a major update to the DrugBank database for 2018. *Nucleic Acids Res*. **46**, D1074-D1082, (2018).

3 Morris, G. M. *et al.* AutoDock4 and AutoDockTools4: Automated docking with selective receptor flexibility. *J Comput Chem*. **30**, 2785-2791, (2009).

4 Trott, O. & Olson, A. J. AutoDock Vina: improving the speed and accuracy of docking with a new scoring function, efficient optimization, and multithreading. *J Comput Chem*. **31**, 455-461, (2010).

5 Schrodinger, LLC. *The PyMOL Molecular Graphics System, Version 1.8* (2015).

6 Kim, Y. *et al.* Broad-spectrum antivirals against 3C or 3C-like proteases of picornaviruses, noroviruses, and coronaviruses. *J Virol*. **86**, 11754-11762, (2012).

7 Jin, Z. *et al.* Structure of M(pro) from SARS-CoV-2 and discovery of its inhibitors. *Nature*. **582**, 289-293, (2020).

8 Ma, C. *et al.* Boceprevir, GC-376, and calpain inhibitors II, XII inhibit SARS-CoV-2 viral replication by targeting the viral main protease. *Cell Res*. **30**, 678-692, (2020).

Figure. S1


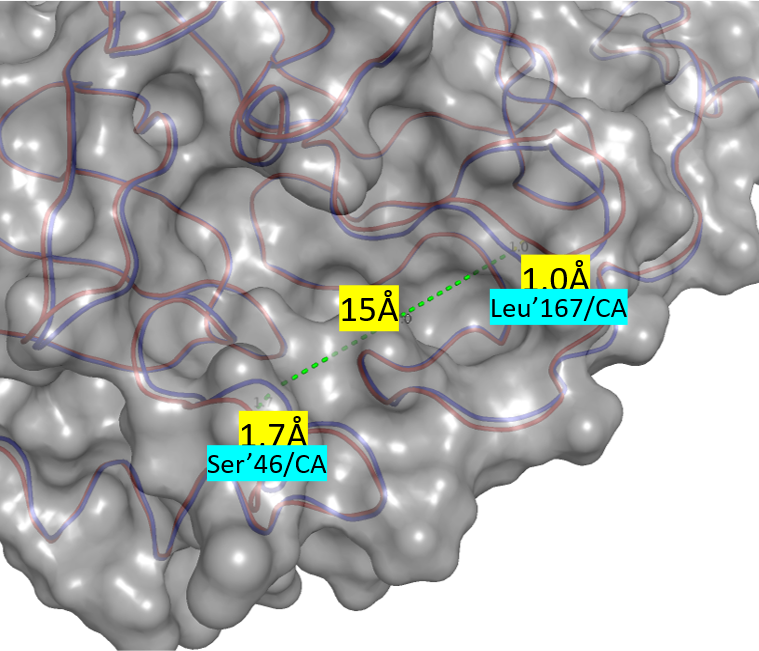


Supplementary Figure. S1. The active pocket of SARS-CoV-2 M^pro^ is flexible. Structure alignment of SARS-CoV-2 M^pro^ 6lu7 (red line) and 6m2q (blue line), the distances between Ser’46/CA and Leu’167/CA were measured.

Figure. S2


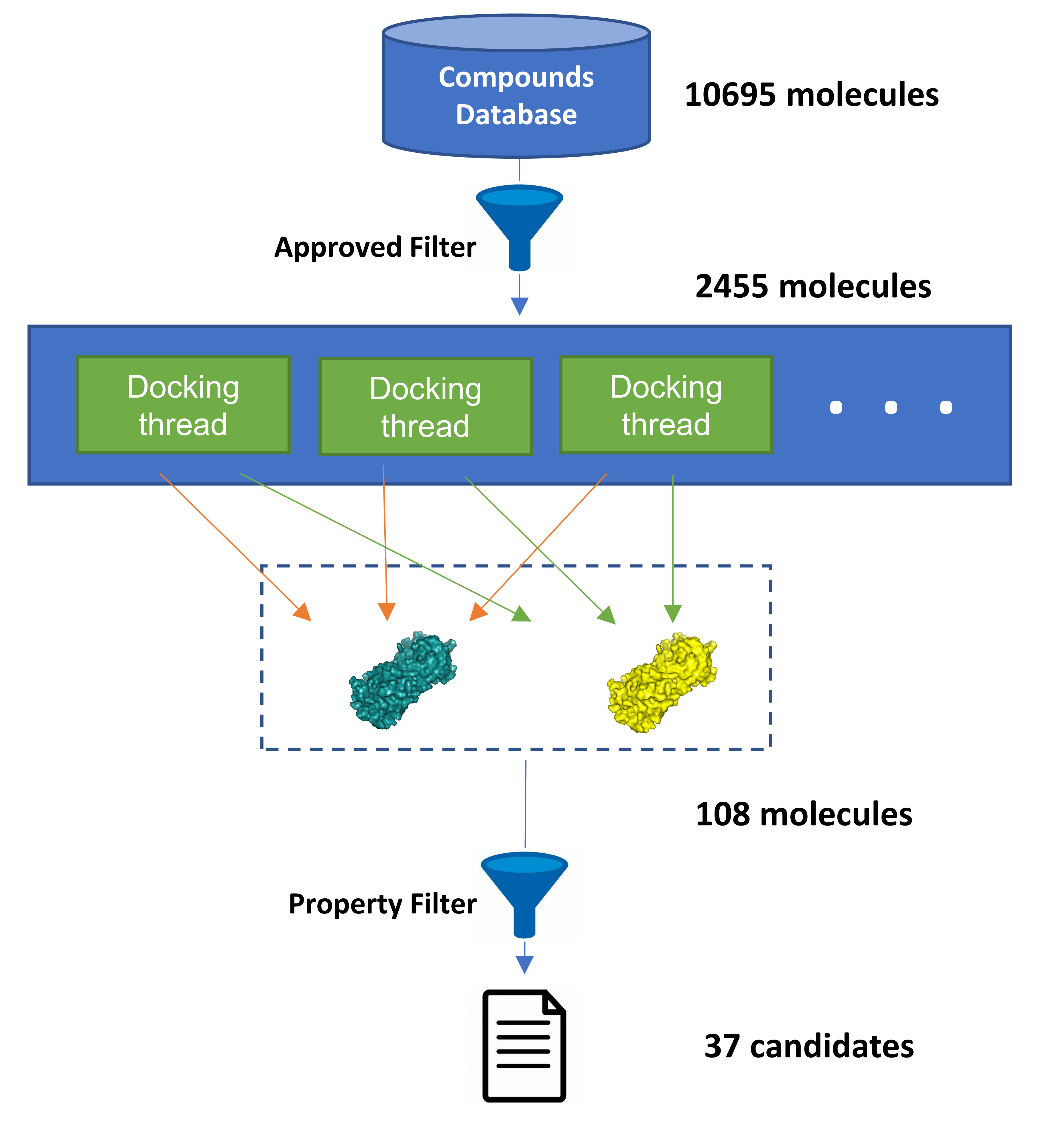


Supplementary Figure. S2. Schematic workflow for virtual screening. Workflow used for virtual drug screening by using a multiple cross-docking strategy based on the Vina program, combined with our in-house multi-thread screening program.

Figure. S3


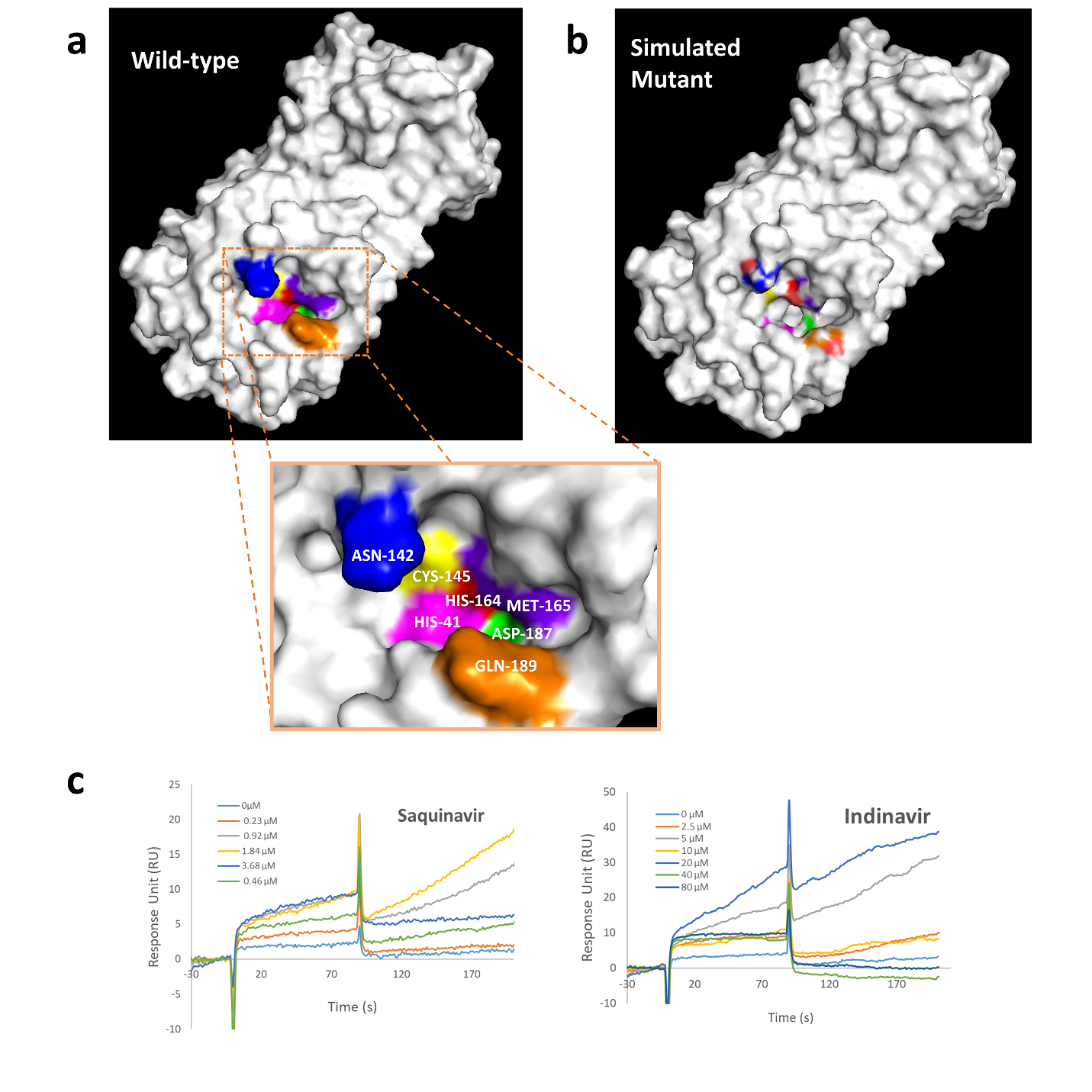


Supplementary Figure. S3. Mutagenesis studies (a) Distribution of residues in the active pocket of M^pro^ (41H in pink, 142N in blue, 145C in yellow, 164H in red, 165M in violet, 187D in green, 189Q in brown). (b) The simulated mutant by mutagenesis analysis after all the residues replaced with Ala. (c) The SPR kinetic plots of the sample drugs interaction with the mutant.

Figure. S4


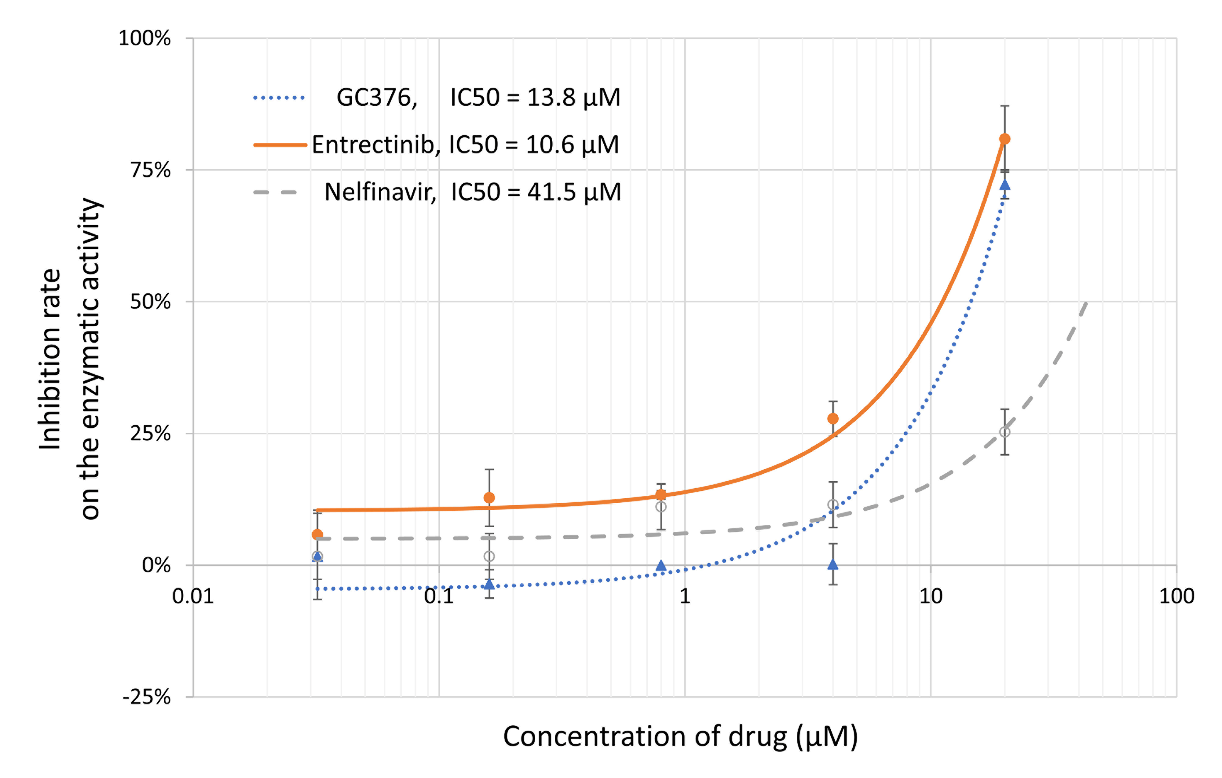


Supplementary Figure. S4. Inhibition curves on the enzymatic activity of SARS-CoV-2 M^pro^. 200 nM M^pro^ was treated with different drugs with a 5-fold concentration gradient from 0.032 µM to 20 µM. Enzymatic activity of the M^pro^ were detected by RFU releases of FRET substrate for 3 h. The inhibition rates were calculated and the fitting curve with a logarithmic scale for the x-axis was drawn, by which the IC50 values of drugs were obtained. Each sample was tested in triplicate.

Figure. S5


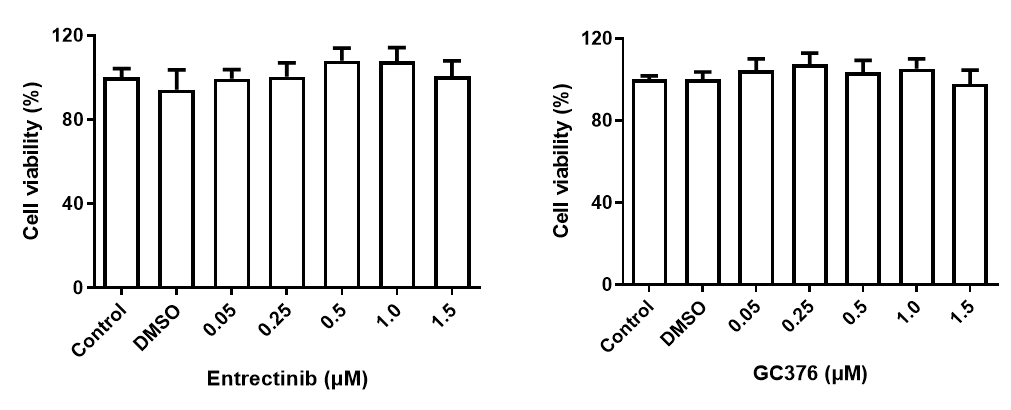


Supplementary Figure. S5. Cytotoxicity assays. Vero E6 cells were seeded at 2×10^4^ per well in 96-well plates (Corning) and incubated at 37℃ overnight. Cells were infected with SARS-CoV-2 (0.01 MOI) for 2h, and incubated with indicated drugs (0.05-1.5 μM) for 48h. After that, cytotoxicity was detected by using CCK-8 according to the manufacturer’s protocol. There was no significant difference between each dose of treatment and the control (P > 0.05), which suggested that no cytotoxicity occurred when the concentration of the drugs was less than 1.5 μM.

Supplementary Table S1. Candidates for M^pro^ identified by virtual screening.

| **Approved Drugs** | **MW (g/mol)** | **Description** | **Average Vina Score (kcal/mol)** |
| --- | --- | --- | --- |
| **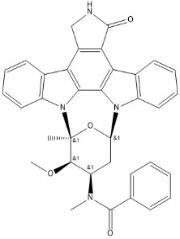**  Midostaurin | 570.6 | A multi-kinase inhibitor with potential antiangiogenic and antineoplastic activities | -10.00 |
| 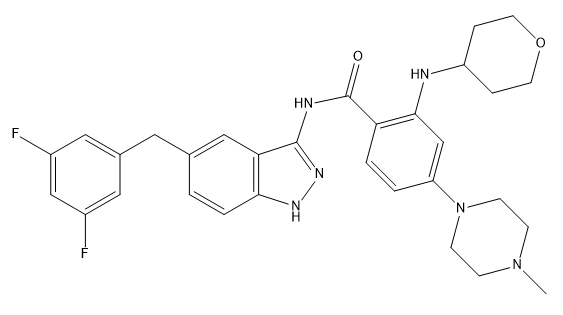  Entrectinib | 560.65 | A kinase (TRK, ROS and ALK) inhibitor for the treatment of relevant non-small cell lung cancer and solid tumors | -9.50 |
| 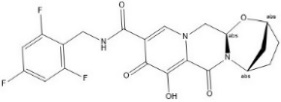  Bictegravir | 449.4 | HIV-1 integrase strand transfer inhibitor | -9.00 |
| 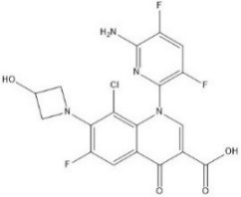  Delafloxacin | 440.8 | A fluoroquinolone antibiotic | -8.90 |
| 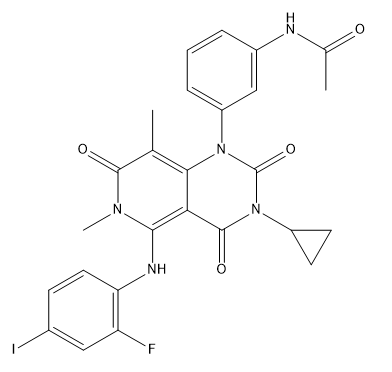  Trametinib | 615.39 | a kinase inhibitor to treat anaplastic thyroid cancer | -8.85 |
| 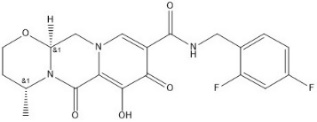  Dolutegravir | 419.4 | HIV integrase strand transfer inhibitor | -8.85 |
| 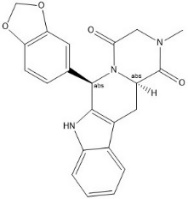  Tadalafil | 389.4 | Inhibitor of cGMP degradation, used to treat erectile dysfunction, pulmonary arterial hypertension | -8.70 |
| 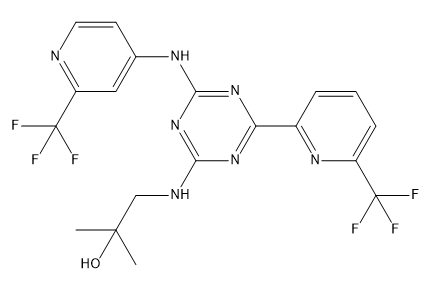  Enasidenib | 473.38 | An allosteric inhibitor of mutant IDH2 enzyme, for the treatment of relapsed or refractory acute myeloid leukemia with IDH2 gene mutations | -8.55 |
| 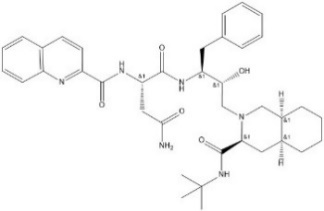  Saquinavir | 670.8 | A HIV protease inhibitor | -8.45 |
| 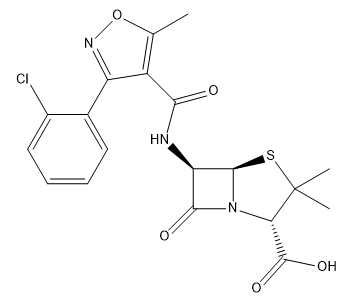  Cloxacillin | 435.88 | A semi-synthetic antibiotic | -8.40 |
| 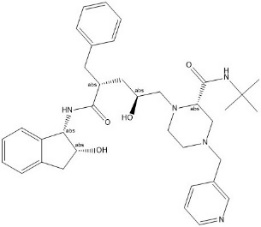  Indinavir | 613.8 | A HIV protease inhibitor | -8.30 |

Supplementary Data S1. Original Docking Data
